# Post-Covid condition and clinic characteristics associated with SARS-CoV-2 infection: a 2-year follow-up to Brazilian cases

**DOI:** 10.1038/s41598-023-40586-8

**Published:** 2023-08-26

**Authors:** Nayara Sousa da Silva, Nathália Kelly de Araújo, Katiusse Alves dos Santos, Karla Simone Costa de Souza, Jéssica Nayara Góes de Araújo, Marina Sampaio Cruz, Esteban J. Parra, Vivian Nogueira Silbiger, André Ducati Luchessi

**Affiliations:** 1 Northeast Biotechnology Network (RENORBIO), Graduate Program in Biotechnology, Federal University of Rio Grande do Norte, Natal, Brazil; 2 Graduate Program of Health and Sciences, Federal University of Rio Grande do Norte, Natal, Brazil; 3 Department of Biological Sciences, Federal Institute of Rondônia, Guajará-Mirim, Brazil; 4 Graduate Program of Pharmaceutical Sciences, Federal University of Rio Grande do Norte, Natal, Brazil; 5 Department of Clinical and Toxicology Analysis, Federal University of Rio Grande do Norte, Av. General Gustavo Cordeiro de Farias, 384, Natal, RN 59012-570 Brazil; 6 Division of Cardiology, Department of Medicine, UC San Diego, San Diego, CA USA; 7 Department of Anthropology, University of Toronto at Mississauga, Mississauga, ON Canada

**Keywords:** SARS-CoV-2, Infectious diseases

## Abstract

Until January 2023, Brazil recorded 37 million COVID-19 cases despite the decrease in mortality due to mass vaccination efforts against COVID-19. The infection continues to challenge researchers and health professionals with the persistent symptoms and onset manifestations after the acute phase of the disease, namely Post-Covid Condition (PCC). Being one of the countries with the highest infection rate, Brazil must prepare for a growing number of patients with chronic health consequences of COVID-19. Longitudinal studies that follow patients over extended periods are crucial in understanding the long-term impacts of COVID-19, including potential health consequences and the effects on quality of life. We describe the clinical profile of a cohort of COVID-19 patients infected during the first year of the pandemic in Brazil and a follow-up after two years to investigate the health impacts of SARS-CoV-2 infection. The first wave of SARS-CoV-2 infection in Brazil featured extensive drug misuse, notably the ineffective COVID kit comprised of ivermectin, antimalarials and azithromycin, and elevated in-hospital mortality. In the second phase of the study, Post-Covid Condition was reported by symptomatic COVID-19 subjects across different severity levels two years after infection. Long haulers are more likely to be women, previously hospitalized, and reported a range of symptoms from muscle pain to cognitive deficit. Our longitudinal study is essential to inform public health authorities to develop strategies and policies to control the spread of the virus and mitigate its impacts on society.

## Introduction

The first case of Severe Acute Respiratory Syndrome Coronavirus 2 (SARS-CoV-2) infection was officially reported in Brazil on February 26, 2020^1^. Since then, public health actions adopted to mitigate the SARS-CoV-2 spread have been unsuccessful, leading to a collapse of Brazil’s health system between 2020 and 2021^2^. Consequently, Brazil had one of the highest case-fatality ratios proportionally to COVID-19 cases, with nearly 37 million confirmed cases and exceeding 697,000 deaths as of January 2023^3^. As the pandemic progressed to a controlled scenario due to the widespread COVID-19 vaccination, many cohort studies revealed symptom persistence and other health impacts due to SARS-CoV-2 infection, drawing attention from researchers and healthcare agencies worldwide^4–6^.

Post-covid Condition (PCC), or Long COVID, is a multisystemic disturbance with symptoms persisting for at least 12 weeks after an acute SARS-CoV-2 infection that cannot be explained by an alternative diagnosis^7^. The World Health Organization estimates that around 20% of individuals infected with SARS-CoV-2 may present some version of PCC^8^. However, these numbers may change considerably depending on the timeline, region, and population studied^6, 9^. The mechanisms underlying the development of PCC and its risk factors have not been fully elucidated. PCC is frequently associated with females and younger individuals, and even though hospitalization during COVID-19 raises the odds of PCC, persistent symptoms are also reported by mild COVID patients^10, 11^.

SARS-CoV-2 infection continues to be an intricate topic. The unknown long-term health impacts after the infection, especially in children and teenagers^12^, might shortly lead to a more significant public health crisis. Brazil is one of the countries most affected by the pandemic due to bad management and the promotion of pretreatment drugs without scientific support, namely the "COVID Kit", an association of ivermectin, antimalarials and azithromycin^13, 14^. Currently, Brazil must prepare for the long-lasting consequences of PCC.

Here, we describe the clinical profile of SARS-CoV-2 infected Brazilians included in the COVID-19 SCOURGE study performed in 2020^15^, as well as the PCC characteristics of the patients after a follow-up two years after the first infection. In the first phase of the study, the “COVID Kit” euphoria and high mortality of hospitalized patients reflect one of the darkest moments of the pandemic in the country. Two years later, the surviving patients still must dwell on persistent symptoms that impact their well-being. To understand the dynamics of the disease in the country, a temporal-focused study could identify the health reality, necessities, and struggles of COVID-19 patients that developed PCC.

## Results

**Figure 1:**
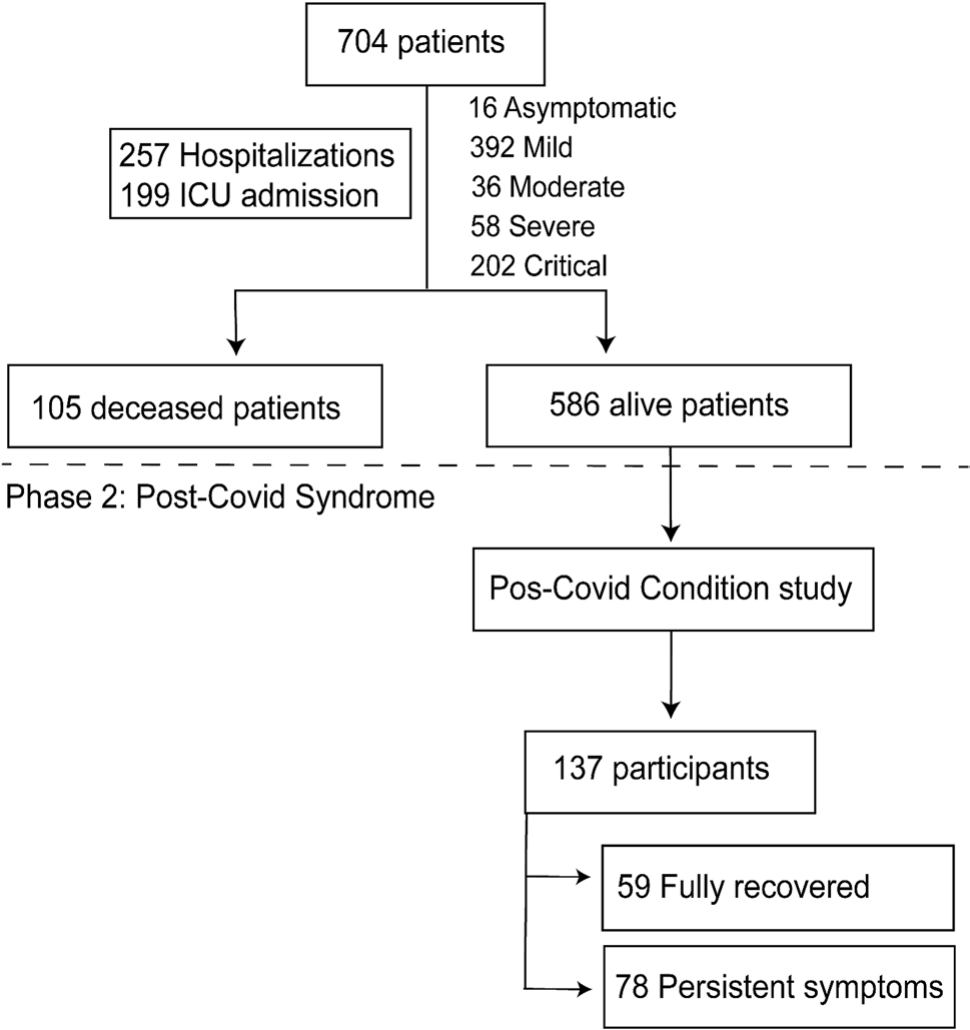
Flowchart depicting the two stages of the study and subjects’ stratification according to the event of interest.

From March to August 2020, 704 patients diagnosed with SARS-CoV-2 infection were enrolled in Phase I of our study. The most common diagnostic assay was PCR (74.2%), follow by serological assays (23.4%). A very small proportion of the sample (17 patients or 2.4% of the sample) was included based solely on a clinical diagnosis. Out of the 704 patients, 257 were hospitalized, 199 required ICU admission, and 105 died. During the study’s second phase, in 2022, we contacted all surviving patients for a follow-up on symptom persistence (Fig. 1).

## Phase I: Acute phase of SARS-COV-2 infection

**Figure 2:**
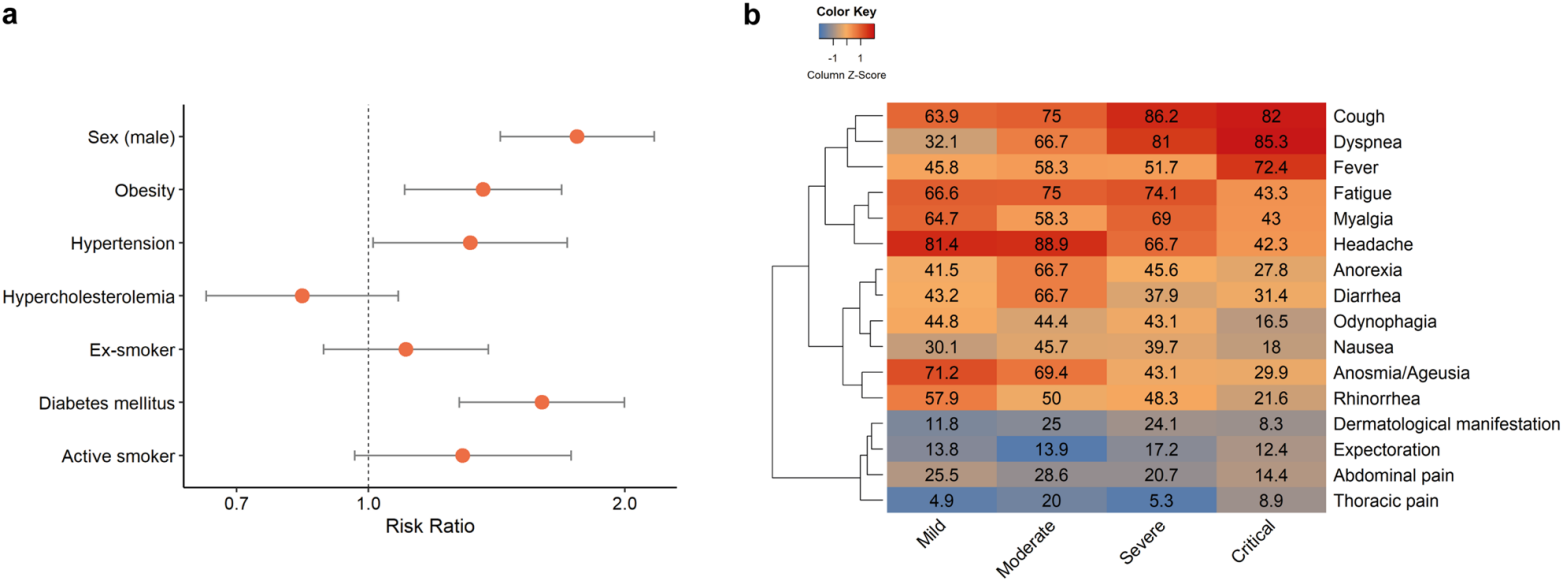
Severity risk factors and symptoms of COVID-19 patients. (**a**) Age-adjusted risk ratio from Poisson robust regression considering mild and critical (reference) cases. (**b**) Heatmap of COVID-19 symptoms, color scaled by column.

Approximately 56% of the subjects were classified as mild COVID-19 cases and 29% as critical cases (Table 1). Mild patients comprised predominantly women, younger individuals, and non-smokers. When adjusted for age, males had a higher risk of having critical COVID-19 (RR = 1.76, 95%Cl: 1.43–2.17, *p* = < 0.001), as well as subjects with hypertension (RR = 1.32, 95%Cl: 1.01–1.71, p = 0.040), obesity (RR = 1.36, 95%Cl: 1.10–1.69, p = 0.004), and diabetes (RR = 1.60, 95%Cl: 1.28–2.00, p = < 0.001), Fig. 2a.

**Table 1:** COVID-19 patients’ characteristics during first infection (2020).

	Asymptomatic	Mild	Moderate	Severe	Critical	*p*-value
	n = 16	n = 392	n = 36	n = 58	n = 202	
Sex, n (%)						
Female	8 (50)	231 (58.9)	21 (58.3)	30 (51.7)	66 (32.7)	< 0.001
Male	8 (50)	161 (41.1)	15 (41.7)	28 (48.3)	136 (67.3)	
Smoker, n (%)						
Non-smoker	16 (100)	349 (89.3)	28 (77.8)	32 (62.7)	112 (64.0)	< 0.001
Ex-smoker	0	30 (7.7)	8 (22.2)	18 (35.3)	53 (30.3)	
Active	0	12 (3.0)	0	1 (2.0)	10 (5.7)	
Age (years), median (IQR)	40.64 (9.5)	37.49 (12.8)	41.44 (13.1)	49.78 (27.1)	63.85 (26.4)	< 0.001
Age stratified, n (%)						
Up to 40 years	8 (50)	248 (63.3)	16 (44.4)	18 (31.6)	25 (12.7)	< 0.001
41–50	7 (43.8)	91 (23.2)	11 (30.6)	12 (21.1)	29(14.7)	
51–60	1 (6.3)	35 (8.9)	7 (19.4)	8 (14.4)	30 (15.2)	
61–70	0	15 (3.8)	0	14 (24.6)	42 (21.3)	
71–80	0	3 (0.8)	2 (5.6)	5 (8.8)	43 (21.8)	
Over 81 years	0	0	0	0	28 (14.2)	
BMI (kg/m^2^), median (IQR)	29.35 (8.1)	26.89 (6.2)	26.79 (5.8)	28.58 (7.6)	27.78 (8.1)	0.058
Underlying medical conditions, n (%)						
Hypertension	4 (25)	79 (20.2)	6 (16.7)	28 (48.3)	133 (65.8)	< 0.001
Hypercholesterolemia	0 (0.0)	34 (8.7)	7 (19.4)	14 (24.1)	23 (11.4)	0.002
Diabetes Mellitus	0 (0.0)	19 (4.8)	3 (8.3)	21 (36.2)	94 (46.5)	< 0.001
Obesity	8 (50)	108 (27.6)	12 (33.3)	25 (43.1)	76 (37.6)	0.017
Cardiac insufficiency	0 (0.0)	3 (0.8)	0 (0.0)	8 (13.8)	23 (9.8)	< 0.001
COPD	0 (0.0)	1(0.3)	0 (0.0)	2 (3.4)	27 (13.4)	< 0.001
Chronic kidney disease	0 (0.0)	9 (19.5)	0 (0.0)	4 (2.9)	22 (10)	< 0.001

Figure 2b displays symptoms of each severity level in clusters. Classic COVID-19 symptoms such as headache, anosmia, and fatigue were reported by over 65% of the mild patients. In moderate cases, gastrointestinal symptoms and respiratory manifestations became more common. With the progression of severity, typical symptoms such as anosmia or ageusia were less frequent, whereas respiratory problems such as dyspnea and cough were experienced by over 80% of individuals. Fever and respiratory distress are the most frequent symptoms in critical patients.

**Figure 3:**
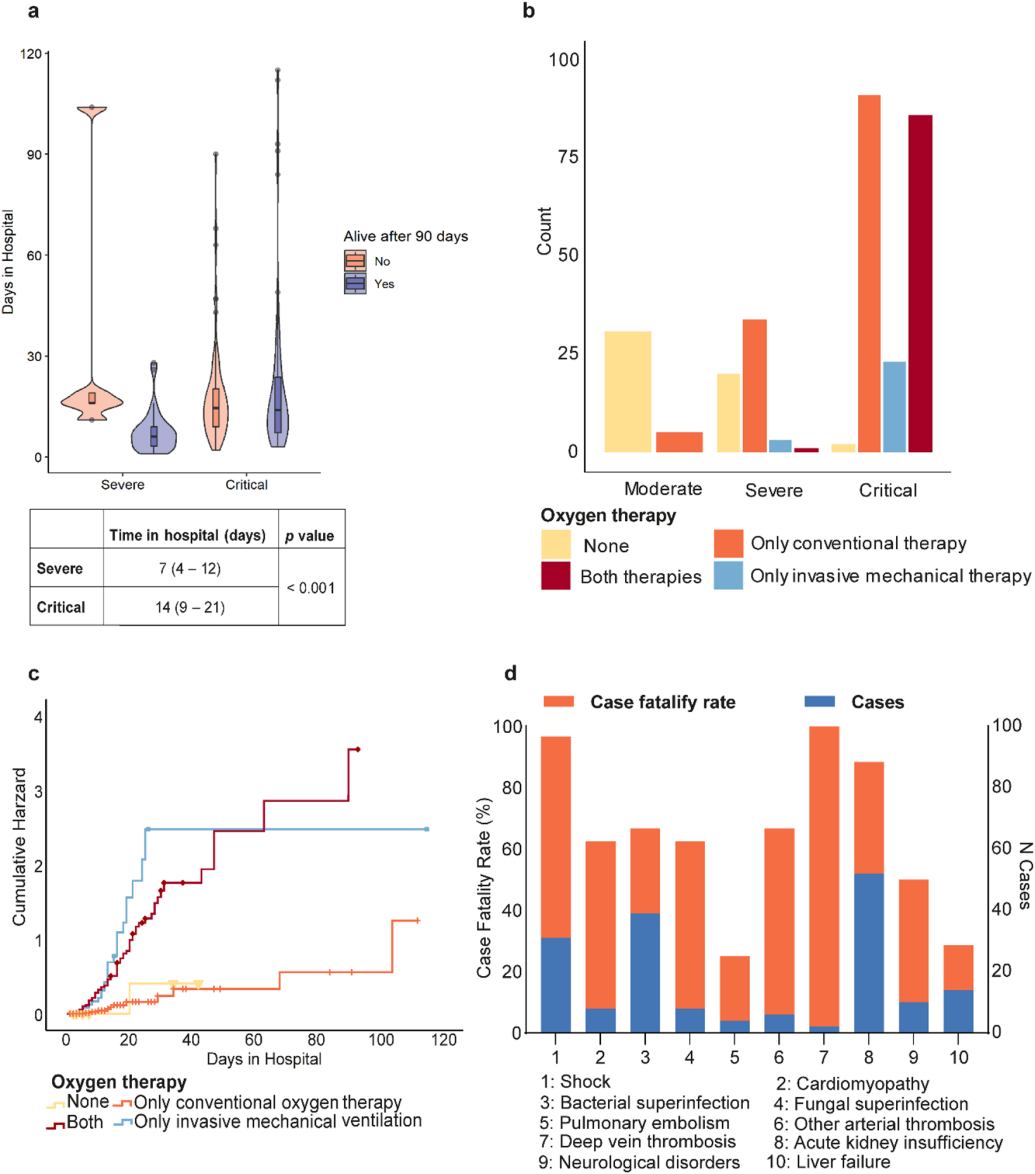
Hospitalization characteristics and pharmacotherapy in COVID-19 patients. (**a**) Hospitalization duration of severe and critical patients stratified by survival. (**b**) Types of oxygen therapies employed in hospitalized patients. (**c**) Cumulative hazard of death versus days of hospitalization according to the type of oxygen therapy. (**d**) Cases and fatality rate of complications from COVID-19.

Hospitalization was necessary for 257 patients, of which ten were moderate, 45 severe, and 202 critical cases. The median time between onset of symptoms and hospital admission was similar across the three groups: 8 (IQR = 4), 9 (IQR = 6) and 8 (IQR = 6), respectively, *p* = 0.438. ICU admission was required for 199 critical patients. Biomarker data from critical patients is provided in Additional file 4. The survival variable was filled after confirmation by phone contact 90 days post-infection; 13 patients were lost in the follow-up. However, we could confirm the death of 105 subjects, five severe and 100 critical cases. Critical patients had a median hospitalization time twice as long as severe patients (Fig. 3a). However, the extent of hospitalization was not associated with survival in this group.

Conventional oxygen therapy was employed in 13.9% of moderate cases, 60.3% of severe cases, and 87.6% of critical cases (Fig. 3b). In critical cases, conventional oxygen therapy was often substituted for invasive mechanical ventilation (IMV) at some point (42.6%). Only 11.4% of patients received invasive mechanical therapy as the first treatment option. The use of IMV outside of ICU settings was observed in some severe patients.

The cumulative hazard rate of death (Fig. 3c) increased dramatically after 20 days in the hospital for patients that received only IVM or both types of oxygen support. After 100 days, 60 percent of patients that received only conventional therapy were alive, in contrast to 10% of patients that received only IMV and 15.1% that received both types. Complications from COVID-19 or hospitalization were common. The most frequent complications recorded were acute kidney insufficiency and bacterial superinfection (Fig. 3d). The most fatal was deep vein thrombosis (100% of affected patients) and shock (96% of affected patients).

**Figure 4:**
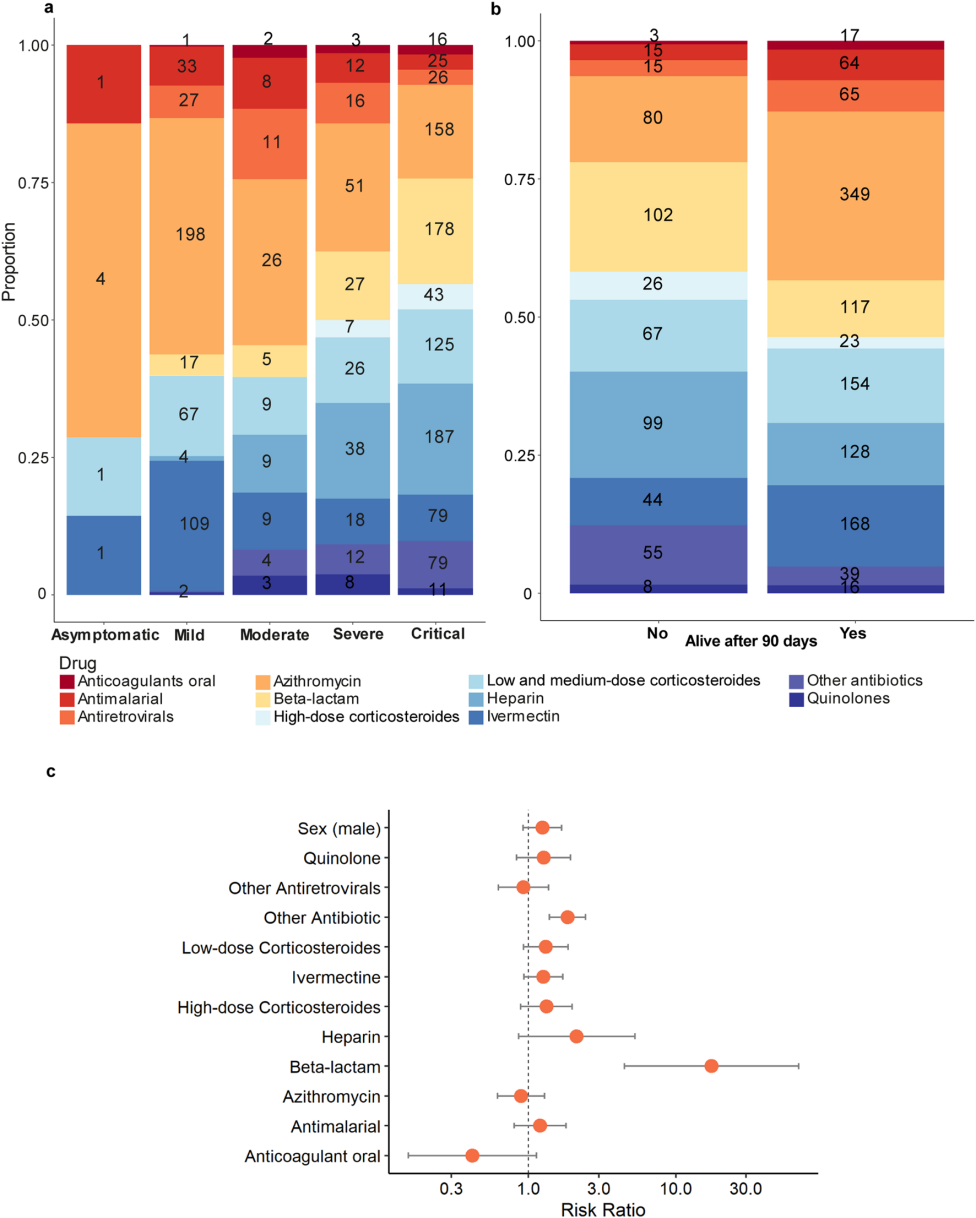
Pharmacotherapy in COVID-19 patients and its risk to mortality (**a**) Drugs used by COVID-19 patients according to severity level. (**b**) Drugs used by critical patients according to survival. (**c**) The risk ratio of drug intake and death in critical patients, adjusted for age and sex.

Pharmacotherapy was reported by subjects across the five levels of severity. Regarding the “COVID Kit,” the intakes of antimalarial (*p* = 0.010), azithromycin (*p* < 0.001), and ivermectin (*p* = 0.010) were higher in severe and critical groups (Fig. 4a). Antimalarial intake was similar across surviving and deceased patients (*p* = 0.406). The frequency of ivermectin intake within the critical group (Fig. 4b) is statistically higher in dead subjects (*p* = 0.008). However, no effect on survival was found by logistic regression (*p* = 0.213). A temporal view of “COVID Kit” intake by patients is provided in Additional file 5. The consumption of Beta-lactams and other antibiotics (Fig. 4c) was associated with higher risk of death in critical patients (beta-lactam: RR = 17.49, 95% CI:4.49–68.12, *p* < 0.001; other antibiotics: RR = 1.84, 95% CI: 1.39–2.44, *p* < 0.001).

## Phase II: Post-COVID condition

During the first phase of our study, 586 subjects survived. The age distribution of these individuals was: 303 (51.9%) up to 40 years, 141 (24.1%) between 41–50 years, 66 (11.3%) between 51–60 years, 42 (7.2%) between 61–70 years, 23 (3.9%) between 71–80 years and 9 (1.5%) over 81 years.

Of the 586 subjects, 137 (23.4%) patients agreed to participate in Phase II of our study. The prevalence of PCC across COVID-19 severity levels was similar. However, it is important to mention that at the end of Phase I of the study, only 93 (41.2%) patients with critical COVID-19 were alive, so the 18 critical subjects interviewed in Phase II only represent ~ 19% of surviving critical patients.

**Figure 5:**
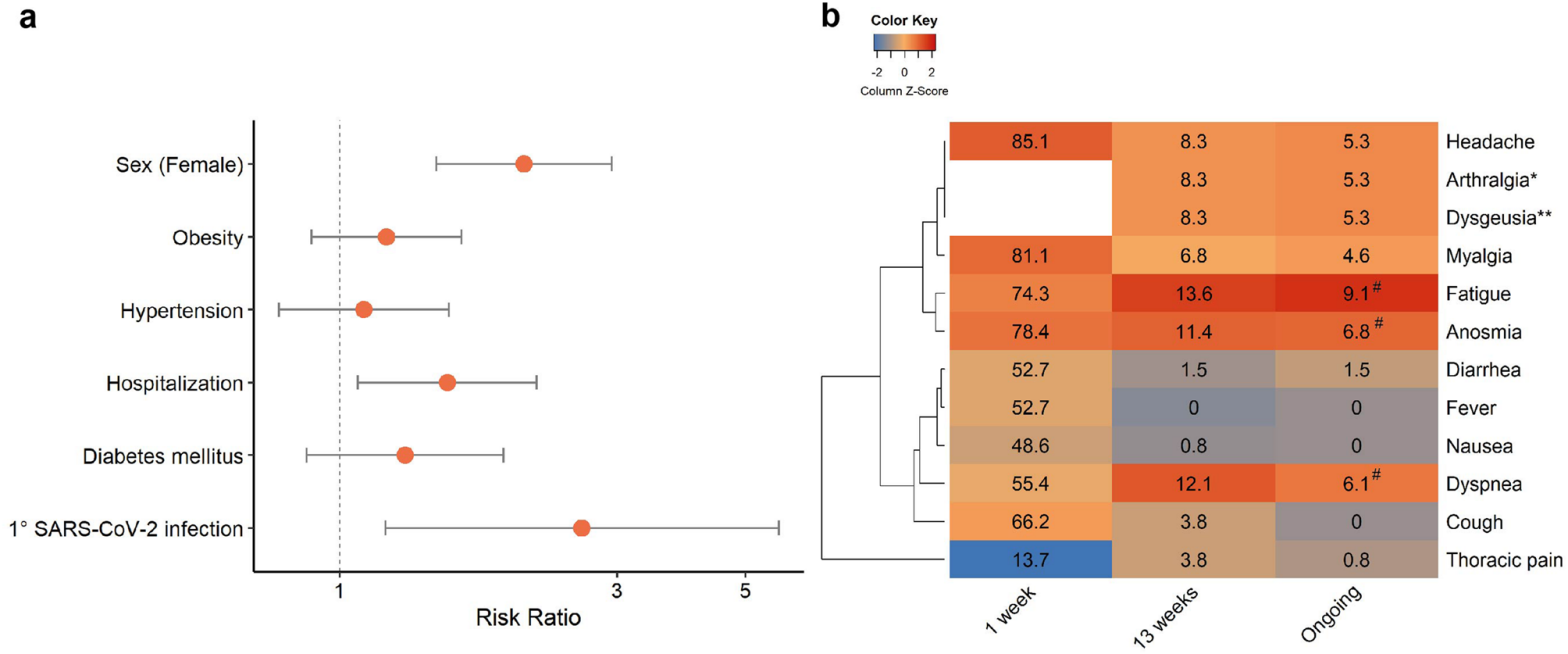
Severity risk factors and symptoms of Post COVID-Condition. (**a**) Risk ratio variables related to the development of PCC. (**b**) Heatmap of PCC symptoms scaled by column. * Arthralgia and myalgia were considered the same variable in COVID-19 patients.  **Dysgeusia and anosmia were considered the same variable in COVID-19 patients. McNemar’s test between “13 weeks” and “ongoing” data *p* ≤ 0.05.

The Phase II sample consisted of 73 (53.3%) women and 64 (46.7%) men. Self-reported persistence and ongoing symptoms or alterations after 13 weeks of infection (e.g., individuals with Post-Covid Condition or PCC) were disclosed by 78 patients (56.9% of the Phase II sample). Table 2 lists the general characteristics of the PCC and non-PCC groups. Sex and hospitalization were significantly associated with PCC in a robust Poisson regression adjusted for age (Fig. 5a). Women have a higher risk of developing PCC, as well as hospitalized patients. Comorbidities such as hypertension, hypercholesterolemia, diabetes and obesity were more frequent in the PCC group than in the non-PCC group, but the differences were not significant (Table 2). Regarding reinfections, 38 patients (27.7%) had at least one confirmed reinfection. However, most of the individuals (94.9%) claimed persistence of the symptoms after the first infection, and only four patients declared persistence of symptoms after a second infection. At the time of the interview, 69.2% of patients reported still experiencing some health conditions after COVID-19, including respiratory disturbance, alteration of smell and taste, and body aches (Fig. 5b). The median duration calculated was 681 days (IQR 69).

**Table 2:** Post-COVID Condition patients characteristics (2022).

	No PCC	PCC	*p*-value
Sex, n (%)			
Female	20 (39.9)	53 (67.9)	< 0.001
Male	39 (66.1)	25 (32.1)	
Age years, median (IQR)	35.89 (10.69)	39.61 (15.68)	0.348
Age stratified, n (%)			
Up to 40 years	38 (64.4)	44 (56.4)	0.348
41–50	11 (18.6)	18 (23.1)	
51–60	6 (10.2)	11 (14.1)	
61–70	2 (3.4)	2 (2.6)	
71–80	1 (1.7)	1 (1.3)	
Over 81 years	1 (1.7)	2 (2.6)	
Comorbidities at time of COVID-19, n (%)			
Hypertension	15 (25.4)	24 (30.8)	0.568
Hypercholesterolemia	4 (6.8)	11 (14.1)	0.269
Diabetes Mellitus	5 (8.5)	13 (16.7)	0.205
Obesity	17 (28.8)	25 (32.1)	0.712
Reinfections, (n %)			
No	41 (70.7)	49 (70.0)	0.932
Yes	17 (29.3)	21 (30.0)	
Infection-related to PCC, n (%)			
1	43 (74.1)	74 (94.9)	0.001
2	15 (25.9)	4 (5.1)	
Hospitalization, n (%)			
Yes	6 (10.2)	18 (23.1)	0.049
No	53 (89.9)	60 (76.9)	
Severity scale, n (%)			
Asymptomatic	4 (6.8)	0	0.104
Mild	43 (72.9)	54 (69.2)	
Moderate	2 (3.4)	5 (6.4)	
Severe	5 (8.5)	6 (7.7)	
Critical	5 (8.5)	13 (16.7)

After 13 weeks of infection, frequently reported symptoms were fatigue, altered perceptions of smell and taste, and shortness of breath (Fig. 5b). McNemar’s analysis indicates that the frequency of symptoms, such as arthralgia and myalgia, were similar after two years of infection. Other reported symptoms include manifestations that were not directly related to the acute manifestations of COVID-19 observed in Phase I of this study. In particular, memory loss (32.1%), hair loss (17.9%), and anxiety (7.7%) were reported at some point by the patients.

**Figure 6:**
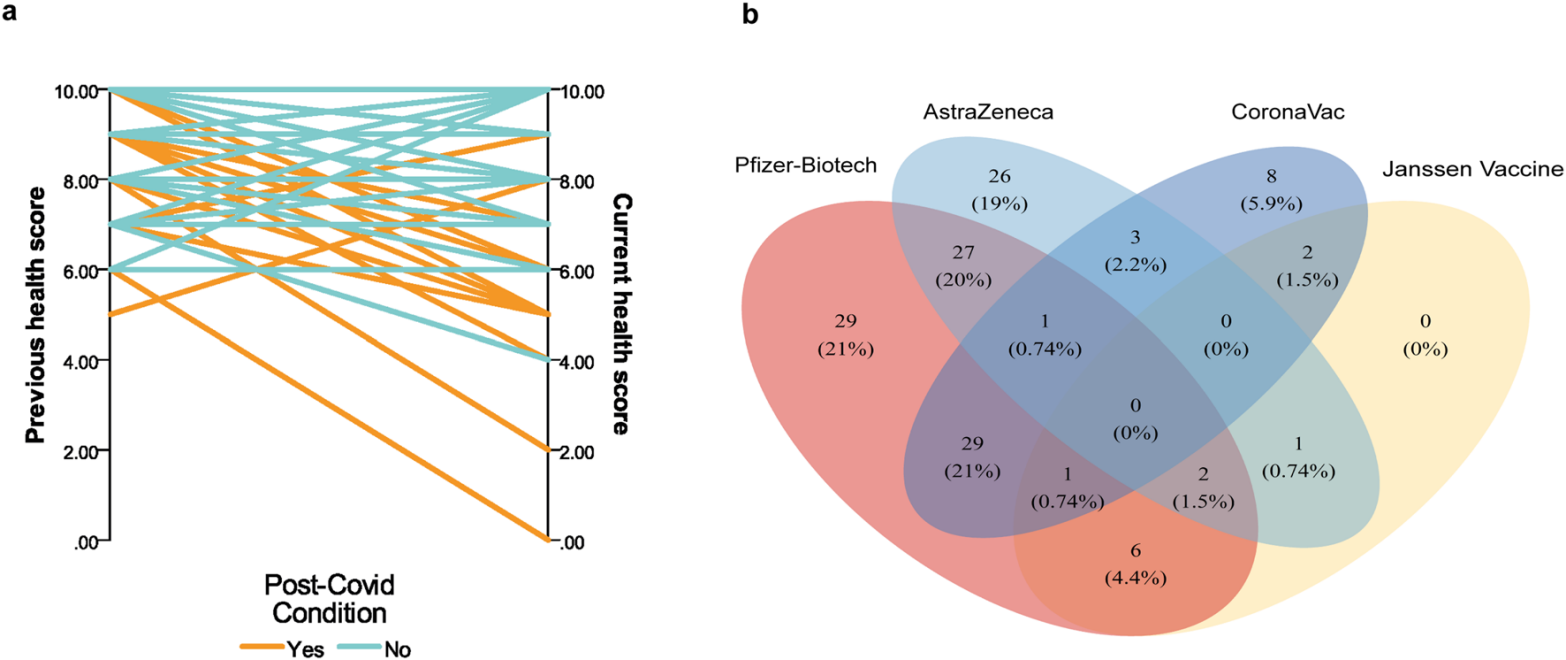
Health and vaccination after COVID-19. (**a**) Parallel diagram of previous and current health perception scores among long haulers and non-long haulers. (**b**) Venn diagram of vaccines reported by subjects on PCC study.

COVID-19 affected the health perception of patients. PCC had an important impact on their daily life activities, and at least 20% of the subjects claimed some level of effort in performing household, work, or social/leisure activities (Table 3). Overall, after infection, long haulers graded their health significantly lower than the previous state (*p* < 0.001), as demonstrated in Fig. 6a. Even though the patients were aware of their condition, only a small percentage had some type of treatment (13 individuals, 16% of the PCC group) and rehabilitation (9 individuals, 11.5% of the PCC group).

**Table 3:** Incapacities in daily life with PCC, results expressed as n (%).

	Personal hygiene	Household activities	Work	Family activities	Social/leisure activities
Unable to perform before COVID-19	2 (2.7)	1 (1.3)	1 (1.3)	1 (1.3)	1 (1.3)
Able to perform as before COVID-19	58 (78.4)	48 (64.0)	49 (65.3)	55 (73.3)	49 (65.3)
Perform with somewhat effort	7 (9.5)	17 (22.7)	16 (21.3)	11 (14.7)	15 (20)
Perform with great effort	7 (9.5)	9 (12.0)	9 (12)	8 (10.7)	9 (12)
Unable to perform anymore, but could do it before COVID-19	0	0	0	0	1 (1.3)

Complete vaccination coverage in our study, with at least two doses, is 93.40%. Of the vaccinated individuals, 69.1% received COMIRNATY (Pfizer-BioNTech), 44.1% Vaxzevria (AstraZeneca), 32.1% CoronaVac (Sinovac Biotech), and 8.8% Janssen Vaccine (Janssen/Johnson & Johnson). The Venn diagram (Fig. 6b) displays the combination of dosages of vaccines. No significant change in PCC symptoms was observed 1 or 3 months after vaccination.

## Discussion

COVID-19 is more than a respiratory infection. The rise of PCC is associated with long-term health effects in patients with different levels of COVID-19 severity. Here, we show how patients that were infected in the year 2020 were dealing with different health impediments after two years of infection.

Phase I of the study began in 2020, during Brazil's first wave of SARS-CoV-2 infection. During Phase I, we recruited a sample of 704 patients classified into five severity levels. The severity risk factors identified in our sample agree with those described in previous studies in the USA, Mexico, Sudan, and China^16–20^. However, the finding of a 1.6 increase in the risk of a subject with diabetes progressing to critical stages of the disease, with respect to individuals without the condition, is alarming. This is particularly concerning given that Brazil ranks fifth among countries with the highest number of people affected by diabetes with a national prevalence estimate of 7.7%^21, 22^.

Symptoms of COVID-19 reported by the patients were broad, indicating the multisystemic response to the infection. The heatmap of symptoms pinpoints the presence of manifestation clusters, which are associated with severity. Mild cases reported a range of symptoms, particularly flu-like symptoms (headache, myalgia, and fatigue) and a high prevalence of anosmia. As severity increases, lesser heterogeneity of manifestations is reported, culminating in a high prevalence of fever, dyspnea, and cough. This does not mean that other symptoms are not present in the severe cases, but rather that they might be underreported. Patients with severe COVID-19 might have less ability to report their symptoms, and also, fever, dyspnea and cough are easily perceived as they are more bothersome and considered alert signals of severity^23^.

The evolution of COVID-19 involves a “tug of war” between viral action and host response. SARS-CoV-2 interacts with ACE2 receptors in organs such as the lungs, heart, gastrointestinal and nervous systems leading to multi-organ disturbance^24^. The host response triggers a pro-inflammatory pathway involving IL-6, INF-γ, and TNF-α. However, in some patients, a dysfunctional response leads to an intense increase in cytokine release, the cytokine storm, leading to septic shock and multiorgan failure^25, 26^. In this regard, the differences in immunological response between sexes have been hypothesized to explain increased severity risk in males^27^.

In our study, 36.5% of the subjects were hospitalized, and within this group of patients, 77.4% were sent to UCI settings at some point. In hospitals, disease progression was monitored by biomarkers, oxygen saturation, and overall vital signs and symptoms. The analysis of biomarkers (see Additional file 4) indicates an intense inflammatory response with liver, endothelia, cardiovascular, and kidney damage. All these disturbances are observed physiologically in the complications reported in Fig. 3d.

Hospitalization duration significantly differs between severe and critical patients. However, the type of oxygen therapy rather than the extent of hospitalization played an important role in survival. Conventional oxygen therapy and IVM were the most common forms of oxygen support. Even though IVM was used as an alternative to alleviate respiratory distress, it is a high-risk procedure with complications such as barotrauma and lung fibrosis^28, 29^. It is not uncommon for patients that survived IVM to need respiratory rehabilitation to overcome muscle weakness^30^. Cases of invasive mechanical ventilation in patients classified as severe (who were not admitted in an ICU setting) were recorded. This atypical situation might have happened during the health collapse due to the lack of infrastructure to accommodate the high number of cases.

Before the availability of COVID-19 drugs, such as Paxlovid, the turmoil surrounding the disease led to the promotion of treatments without scientific proof; at the time, antimalarials, ivermectin, and azithromycin, also called “COVID Kit,” were widely recommended. The use of drugs by asymptomatic and mild patients was seen as “preventive” or as “early treatment.” At least 50% of the mild patients used azithromycin, 28% ivermectin, and 8.4% Antimalarial drugs (Hydroxychloroquine or Chloroquine). In the critical group, the reported use was 78.2%, 40.6% and 12.4%, respectively. However, neither ivermectin nor antimalarials were associated with a favorable outcome within critical patients. Additional file 5 displays the drug intake per diagnosis date. The rush for those drugs started in the first semester of 2020, leading to scarcity and pressuring the government to remove those drugs as over-the-counter^31, 32^. Despite the drop in consumption, the “COVID Kit” continued to be prescribed by physicians in Brazil, with governmental support^13^. It is important to notice that this non-rational drug use carries risks. Cardiotoxicity and other toxic effects were reported during the pandemic in patients that consumed ivermectin and antimalarial in acute high doses and chronically^33, 34^.

Although some authors have observed a correlation between corticosteroid use and survival^35, 36^, no statistical significance was found in our study. We observed that antibiotic intake increased the risks of death in critical patients. This association might have occurred as a secondary effect, as long periods in ICU settings and a compromised immune response increase the risk of infection^37^. Furthermore, increased prescription of antibiotics for COVID-19 patients in ICUs without clear evidence of bacterial co-infection was previously disclosed^38^.

Unfortunately, a lack of infrastructure and effective public policies to deal with a novel virus resulted in Brazil being one of the countries most severely impacted by the COVID-19 pandemic^13, 39^. At the end of the first stage of this study, 105 subjects did not survive, representing 15.0% of the entire study sample and 52.8% of ICU-admitted patients. This high ICU mortality was similar to that in Northeastern Brazil^40^.

Despite the recent drop in cases and death related to COVID-19, SARS-CoV-2 infection is still a concern for health authorities. The recovery time of COVID-19 is still unclear, with some patients fully recovering after some days while others struggle with health complications for months or years^41, 42^.

Of the 586 surviving individuals, we reached nearly 20% of the subjects in Phase II of the study in 2022. More than 50% of the subjects interviewed reported symptom persistence after at least 13 weeks, with 94% linking PCC with the first infection. PCC was disclosed by subjects in all severity levels, except for the asymptomatic group. Women and subjects hospitalized due to COVID-19 were more likely to develop PCC. We could not find a significant association between PCC and age or comorbidities. However, it is important to note that the Phase II sample, comprising 137 individuals, is relatively small. This may reflect a lack of statistical power to identify differences between the PCC and non-PCC groups.

The mechanisms involved in PCC development are still unclear. Some of the suggested hypotheses refer to a compensatory anti-inflammatory response syndrome to counteract the intense inflammatory response to COVID-19^43^ and autoantibodies production^44^. Previous research has highlighted possible risk factors for PCC development, among which female sex appears to be consistent across studies^10, 41, 45–47^. However, there is conflicting information on the impact of some variables on PCC, as seen in the example of age and hospitalization.

Some authors reported a higher likelihood of PCC in younger individuals (< 60 years)^9, 48, 49^, similarly to our study, while increased age was identified as a strong predictor of PCC in other studies^10, 50^. Differences in study design might impact these findings, but more importantly, PCC primarily occurs in patients who have survived the acute phase of COVID-19, meaning that older patients with severe comorbidities had a higher risk of death during the acute phase^51^.

It is well-established that PCC is observed across all levels of severity following a COVID-19 infection. In our study, a similarly high PCC proportion was found after two years of SARS-CoV-2 infection in both hospitalized and non-hospitalized patients (59.7% vs 67.5%), indicating that COVID-19 severity might not be a risk factor for PCC development^23^. However, a meta-analysis including 265,466 patients from 8 studies found that hospitalized patients had more than twice the risk of developing PCC^51^.

The association between hospitalization and PCC needs further investigation, as hospital admission is a broad variable that does not capture the complexity of other in-hospital factors and their interactions that might contribute to a greater risk of PCC development, such as biomarker levels and oxygen therapy. Recently, an association was demonstrated between PCC development and longer time gaps from symptom onset to hospitalization, hospitalization duration, and ICU admission^48^. Another limitation of PCC studies in ICU-admitted patients is the possible confounding effect of post-intensive care syndrome, a known detrimental disorder with physical, mental, and cognitive impairments after ICU discharge^52^.

Of the typical COVID-19 manifestations, fatigue, anosmia, and dysgeusia were commonly reported after 13 weeks of infection, but the frequency of these symptoms was statistically reduced after two years. The non-significant change in proportions of myalgia and arthralgia indicates the persistence of these symptoms in their ongoing lives (Fig. 5b). The reduction of symptoms over time and the long persistence of joint and muscle pains have also been described in previous studies six months and two years after infection^6, 23, 47^.

When asked about symptoms persistence, a few subjects described worsening of their general health caused by the aggravation of gastritis, frequent sore throat, or onset of anemia, diabetes, and hypertension. Long haulers also declared difficulties in memorization (32.1%) and anxiety (7.7%) at some point after the disease. The extent to which many of these symptoms are directly caused by SARS-CoV-2 infection is still unclear. Hyperglycemia after COVID-19 has been reported with many possible triggering mechanisms besides the direct role of SARS-CoV-2^53^. The mental health issues after COVID-19 seem strongly correlated with external factors^54^. However, pathological effects trigged by (or caused by) SARS-CoV-2 might cause cognitive impairments, such as memory loss, concentration deficit, and disfluency^42^. Recently a study described cellular dysregulation related to cognitive impairment after mild COVID-19^55^.

Altogether PCC directly impacted the quality of life of the subjects in our study. Health self-assessment indicated qualitative perception change in general health before and after COVID-19 infection. Our study found a significant reduction in this score, with long haulers reporting an inability to perform daily household activities, work, and social/leisure activities. The decline in life quality after a COVID-19 acute event was also demonstrated by the use of validated questionnaires, in particular regarding mobility, usual activities, anxiety/depression and pain/discomfort^48^.

As of February 2023, 82% of Brazilians are fully vaccinated against COVID-19, according to a global database of COVID-19 vaccination^56^. It has been estimated that more than 300,000 deaths were avoided after the first year of the COVID-19 vaccination program in Brazil^57^. The available COVID-19 vaccines in the country at the time of the study were Vaxzevria (AstraZeneca), CoronaVac (Sinovac Biotech), COMIRNATY (Pfizer-BioNTech), and Janssen Vaccine (Janssen/Johnson & Johnson). These vaccines were integrated into the national vaccination plan at different times. Vaxzevria was the most administered as a primary vaccine (first and second doses) at the national level, and it was predominantly used in individuals aged 40–59 years. COMIRNATY was the most frequently used booster and primary vaccine for younger individuals. Additionally, CoronaVac was employed as the primary vaccination for individuals aged 20–29 years and those above 70 years. The Janssen Vaccine was used in only 4% of all administered doses from 2021–2022^58^ . In our study, COMRNATY was the most common vaccine, followed by Vaxzevria, CoronaVac, and Janssen. Overall, the vaccination profile in our study aligns with the broader national trends, but some variations can be attributed to temporal factors and the characteristics of our study population. Over 90% of the subjects in our study had received at least two shoots, but no significant differences in PCC symptoms after 3 or 6 months of vaccination were found.

The COVID-19 pandemic unfolded into a complex health problem, and Brazil’s response has been widely described as unsuccessful^39^. The general prevalence of PCC is uncertain, but considering the 10–20% proportion suggested by WHO^59^, roughly 7.4 million people could potentially have PCC in Brazil. With mortality controlled by vaccination, the morbidity caused by the PCC should be considered to guide the allocation of investments in public health policies and research investment.

Here, we show the patients’ profiles in two stages of the COVID-19 pandemic in Brazil. In 2020, a higher risk of a SARS-CoV-2 critical infection was associated with males, older individuals, and subjects with comorbidities. During that year, the hype of failed “early treatment” drugs, bad management, and elevated in-hospital mortality placed Brazil in the tragic rank of countries with the worst pandemic indicators^14^. In 2022, patients that survived COVID-19 still experienced long-lasting symptoms, with some ongoing cases after two years of infection. Patients reported PCC across the symptomatic severity levels, and a higher risk was observed in women and previously hospitalized individuals. Long haulers disclosed broad PCC symptoms ranging from muscle and joint pain, cognitive impairment, fatigue, and hair loss. At least 20% of our study sample declared some effort in performing daily activities. Altogether, besides being a frequent condition, assertive policies and actions must be taken to acknowledge those patients and diminish the stigmatization by society and healthcare professionals^60, 61^.

The availability of complete clinical profiles from patients during the first wave of COVID-19 in Brazil and the follow-up after two years to evaluate PCC prevalence and characteristics in this population are two strengths of our study, which provide fascinating insights about PCC in Brazil and the current struggles of long haulers. Our study also has some limitations. This longitudinal prospective cohort study has data from two-time points after COVID-19 infection. Phase I of the study, performed in 2020, was a retrospective analysis subject to the drawbacks of a medical record review which may present incomplete data about the patient's medical history. The loss of follow-up in Phase II of the study, which took place in 2022 to investigate PCC prevalence and characteristics, impacted our sample size, with a consequent loss in statistical power. Phone and message surveys might implicate a selection bias, and the reliance on self-reported symptoms may lead to recall bias^23^. We cannot exclude the possibility of information bias, as other underlying health issues or indirect consequences of SARS-CoV-2 infection might also contribute to detrimental manifestations. However, until standard tools for PCC diagnosis become available, we believe that self-reporting reflects individuals' experiences. Future studies using a more robust validation process to differentiate PCC from other health conditions are needed to mitigate potential bias. Unfortunately, we could not perform any laboratory exams to verify and compare inflammation markers to COVID-19 data.

In conclusion, the SARS-CoV-2 infection remains a significant global health challenge. Brazil’s response to the pandemic in 2020, which relied heavily on unproven preventive drugs, was disastrous. The use of the COVID kit did not improve the survival chances of patients, and the country’s unpreparedness led to one of the worst COVID-19 scenarios worldwide. The COVID-19 challenge now lies in addressing the long-term health and social impacts of COVID-19 survivors who are grappling with PCC of unknown duration. Countries must prepare for the potential new face of COVID-19 by developing clinical protocols to address and recover those new patients. Additionally, further studies are needed to understand the mechanisms and long-term consequences of COVID-19, which will be critical to developing effective diagnostic tools and interventions in the future.

## Methods

### Subjects

Adult patients (> 18 years) with COVID-19 were recruited through social media posts and screened in three major hospitals in Rio Grande do Norte, Brazil (Giselda Trigueiro Hospital, São Lucas Hospital, and Rio Grande Hospital), from March to July 2020. All subjects had a confirmed COVID-19 diagnosis by a positive reverse transcription-polymerase chain reaction (RT-PCR), serological assay or undisputable clinical diagnosis, and signed an approved written informed consent before enrollment.

Data were collected using a standard questionnaire at two time points (see Additional files 1 and 2). In 2020, the questionnaire was COVID-19-focused and consisted of demographic data, pathological history, symptoms, biochemical findings, clinical management, complications, and treatments.

The subjects were categorized into five levels of COVID-19 severity, from asymptomatic to critical. The classification algorithm, described in detail in Additional File 3, is based on NIH guidelines^62^. Briefly, the key clinical criteria were symptoms, lung damage, hospitalization, changes in oxygen saturation, mechanical ventilation, and intensive care unit (ICU) admission. After 90 days of the first contact, a follow-up was performed to confirm the subject’s survival or death.

Between May and June 2022, a PCC-focused questionnaire was applied. This second questionnaire consisted of SARS-CoV-2 infection information, persistence of symptoms, health assistance, health self-assessment, and vaccination. Both questionnaires were produced by the SCOURGE consortium and adapted to the Brazilian reality^15^. The study was approved by the ethics committee of the Onofre Lopes University Hospital (HUOL) under protocol number 31739520.9.1001.5292.

### Data collected

Data were collected from medical records using Research Electronic Data Capture (REDCap) tools hosted at the Centro de Investigación Biomédica en Red (CIBER). To ensure the reliability of data collection, everyone involved in this collection underwent training. In case of uncertainty about the accuracy and reliability of the data, the investigators reviewed the information. All the private information was kept confidential.

### Statistical analysis

Data were analyzed using SPSS version 26.0 (IBM Corporation, Armonk, NY, USA), and all graphs were built using RStudio version 4.2.1 (RStudio Team, Boston, Massachusetts, EUA) and GraphPad Prism version 8 (GraphPad Software, La Jolla, California, USA). Continuous variables were expressed as mean [standard deviation (SD)], or median [interquartile range (IQR)], and categorical variables were presented as frequencies (percentages). The distribution of variables was analyzed using the Kolmogorov–Smirnov test. Variables with normal distributions were subjected to Student's T-Test, and those skew-distributed were analyzed by Mann–the Whitney U test. Differences between categorical variables were tested by χ2 analysis or Fisher's exact test. Cumulative hazard plots were generated using the Kaplan–Meier estimator. P-values < 0.05 were considered significant. Robust Poisson regression was used to evaluate the association of relevant variables with severity (mild vs. critical cases), survival (alive vs. dead after 90 days), and PCC (presence or absence of prolonged symptoms), which is described as risk ratio and 95% confidence intervals. Sex and age were used as covariables.

### Supplementary Information

Supplementary Information 1.

Supplementary Information 2.

Supplementary Information 3.

Supplementary Information 4.

## Data Availability

The data presented in this study are available from the corresponding author on reasonable request.
