# Supplementary material for: Post-Covid condition and clinic characteristics associated with SARS-CoV-2 infection: a 2-year follow-up to Brazilian cases
Source: Sci Rep. 2023 Aug 26;13:13973. doi: 10.1038/s41598-023-40586-8 (PMC10460396; doi:10.1038/s41598-023-40586-8)
Supplement: Supplementary file 4 — Supplementary Information 4. [file 41598_2023_40586_MOESM4_ESM.docx]

**
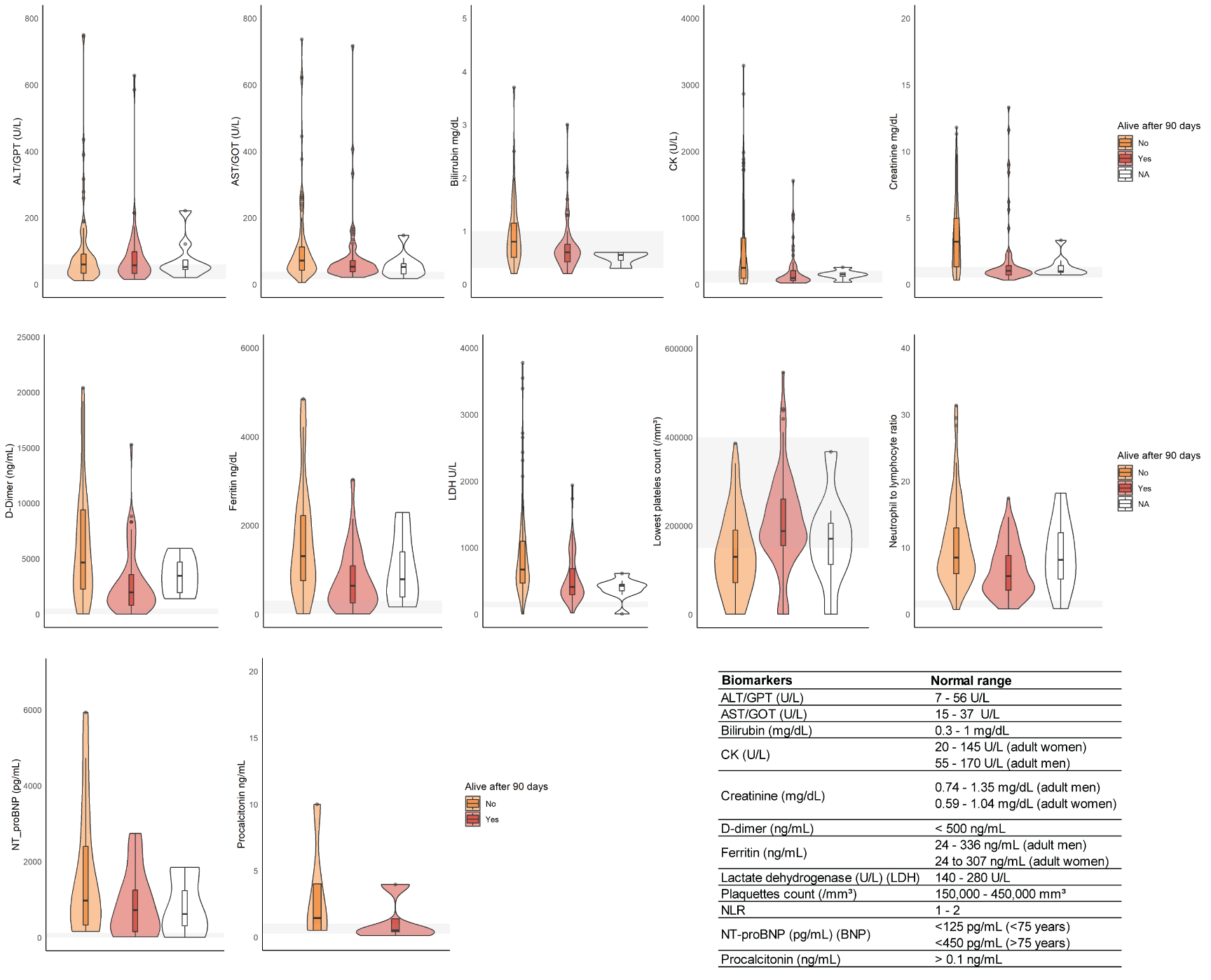
**

**Additional file 4**: Panel with biomarkers levels of critical COVID-19 patients stratified as alive or deceased after 90 days. The typical range results for each biomarker are displayed as a gray bar in graphs and numerically in the table.


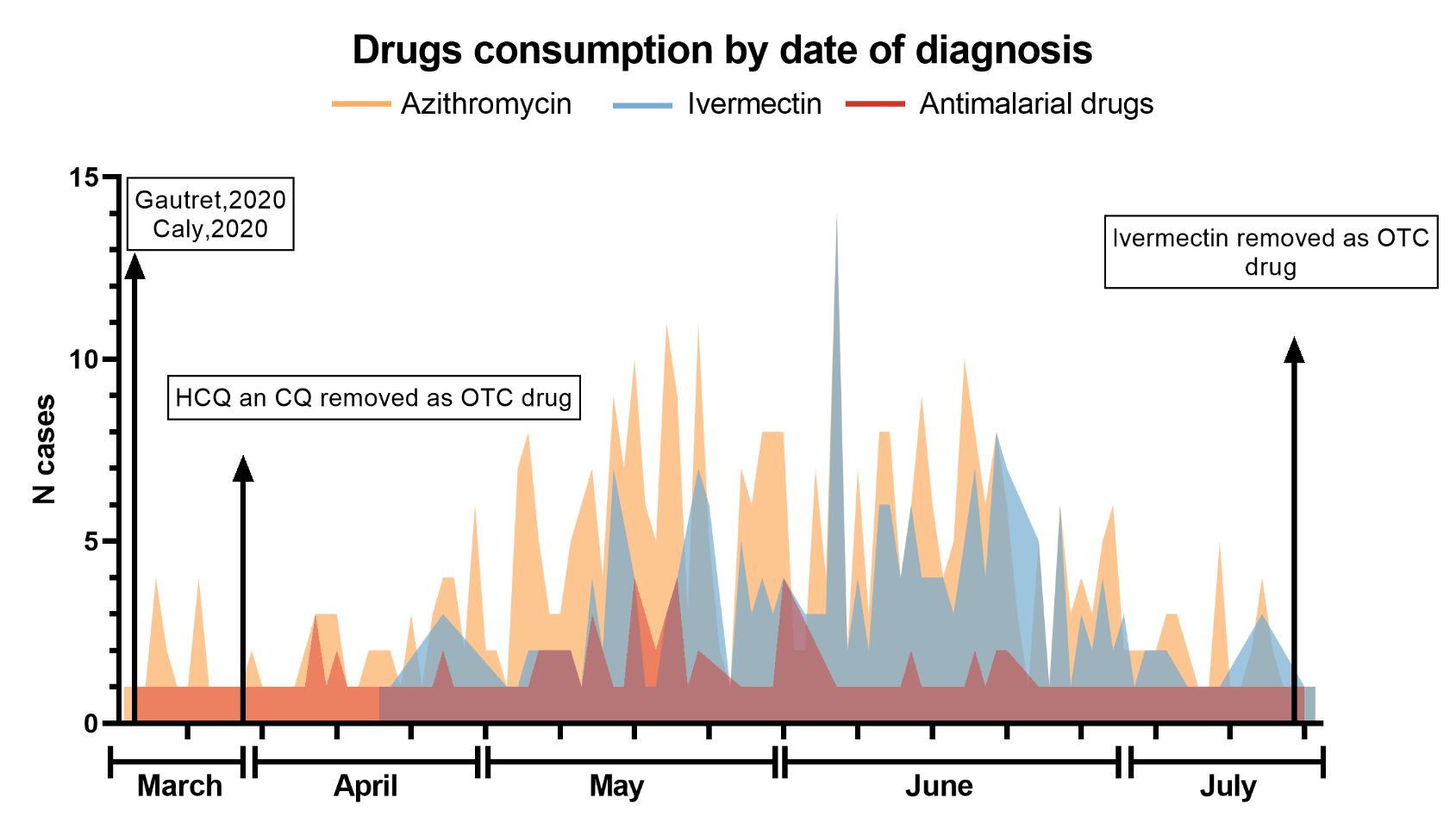


**Additional file 5**: COVID Kit consumption among subjects by diagnosis date. Relevant time points are identified, such as the month of publication of the works from Gautret, 2020 and Caly, 2020, which are the first publications regarding a possible action of ivermectin, antimalarials and azithromycin on COVID-19.
